# Co-occurrence of heavy metals and antibiotics resistance in bacteria isolated from metal-polluted soil

**DOI:** 10.5620/eaht.2023024

**Published:** 2023-11-17

**Authors:** Oluwarotimi John Joseph, Gbemisola Elizabeth Ogunleye, Kubrat Abiola Oyinlola, Augustina I. Balogun, Damilola Tolulope Olumeko

**Affiliations:** 1 Department of Biological Sciences, Faculty of Applied Sciences, KolaDaisi University, Ibadan, Nigeria; 2 Department of Microbiology, Faculty of Science, University of Ibadan, Ibadan, Nigeria

**Keywords:** Heavy metals, Antibiotics, Antibiotic resistance, Metal resistance, *Pseudomonas* sp

## Abstract

The indiscriminate deposition of metal-containing substances into the environment contributes significantly to high concentrations of metals in the soil resulting in resistance to metals and consequentially to antibiotics by inherent microbes which may eventually spread to other pathogenic microbes thereby elevating disease burden due to antibiotic resistance. The study aimed at determining the co-occurrence of resistance of bacteria isolated from metal-contaminated soil to heavy metals and subsequently, antibiotics. Metal-tolerant bacteria were randomly isolated from top soils from a battery waste site using the pour plate method. Selected isolates were identified using biochemical tests, then, subjected to elevating supplemented concentrations of different metal salts at 100-500 μg/mL to determine the minimum inhibitory concentration. Isolates tolerant to minimum three metals up to 400 μg/mL were subjected to Sulfamethoxazole-trimethoprim (25 μg), Imipenem (10 μg), Amoxicillin (30 μg), Ciprofloxacin (10 μg) and Tigecycline (15 μg) and observations interpreted using the guiding principle of the Clinical and Laboratory Standards Institute. Metal concentrations in the soils exceeded permissible limits. In total, 16 isolates were selected and identified as *Proteus* sp. (1), *Pseudomonas* spp. (5), *Enterobacter* spp. (2), *Klebsiella* spp. (2), *Escherichia* spp. (3), *Raoultella* spp. (2) and *Rahnella* sp. (1). Thirteen (81.25 %) of all isolates showed multi-resistance to the metals and seven exhibited multidrug-resistance, with 4 (57.1 %) showing resistance to three different classes of antibiotics and 3 (42.9 %) showed resistance to four antibiotic classes. Heavy metal-tolerant bacteria isolated from this study possess co-selection potentials as they showed resistance to different metals and antibiotics classes which is a concern to public health.

## Introduction

In a bid to improve wellbeing, humans engage in several earth exploration activities such as mining, modern agricultural practices as well as other industrial engagements which have resulted in severe environmental pollution [1-2]. The pursuit for development by humans is a major factor that has directly or indirectly contributed to pollution of the environment. The pollution of soil, air and water bodies by metals has been a great cause of concern in several industrialised nations globally as a result of anthropogenic activities and natural events, thereby resulting in deterioration of the environment. There are evidences that the release of metals into the environment by virtue of human activities become accumulated in the soil in high concentrations with majority arising from industrial sources [3].

However, the movement, toxicity and bioavailability of metals are affected due to their presence in sediments and thus make them available in insufficient concentrations to estimate the environmental effect of polluted sediments [4]. Heavy metals encountered on a daily basis include chromium, manganese, iron, copper, arsenic, silver, cadmium, lead, mercury, nickel and zinc. Thus, the significant phase of metal pollution in water bodies is sediments [5]. Amongst these pollutants, lead (Pb), arsenic (As), nickel (Ni), manganese (Mn), aluminium (Al) and chromium (Cr) are considered hazardous, thus pose serious environmental threats and concerns for human, animal and plant health [6-9].

Meanwhile, due to the persistent exposure of soil microorganisms to concentrations of heavy metals, they become resistant to these metals which are often known to be inhibitory and toxic to these microbial forms. As a result, this resistance selects these microorganisms for resistance to antibiotics, a phenomenon described as “co-selection”. Co-selection refers to a term that describes the ability of a microorganism to exhibit resistance to two or more antimicrobials usually due to coupling of the resistance mechanisms [10].

The spread of resistance to antibiotics amongst autochthonous environmental bacteria in several natural ecosystems has been impacted by the combination of antibiotics and heavy metals pollution [11]. In a study by Berg et al. [12], in an agricultural soil amended with copper resulted in development of resistance to copper by the inherent bacteria which further resulted in co-selection for resistance to multiple antibiotics including chloramphenicol, tetracycline and ampicillin. In addition, bacterial resistance to chloramphenicol and ampicillin was driven by contamination of soil with concentrations of nickel and cadmium [13].

According to numerous reports [11-12], [14-15], environmental contaminants including metals, biocides, and organometallic compounds may also contribute to antibiotic resistance via promoting the spread of mobile genetic elements (MGE) through co-selection. Additionally, Bednorz et al. [16] reported that metal pollution may be a major selector of antibiotic resistance. Noteworthily, in many cases, the copiousness of genes conferring antibiotics resistance is directly associated with degree of environmental metal pollution [17-18].

Antibiotics resistance refers to the state of a microorganism that renders antibiotics inactive against them. Antibiotics resistance in bacteria may be as a result of several factors, majorly the overuse or misuse of antibiotics as well as indiscriminate disposal into the environment. However, the presence of the other pollutants such as heavy metals in the environment, may predispose microorganisms to development of resistance to other forms of antimicrobials such as antibiotics through co-selection which is proving to be a growing concern for the environment and public health [19].

Heavy metals have over the years been used as antimicrobials in fertilizers and other agricultural products and the continuous exposure of soil microorganisms to increased concentrations of heavy metals may predispose them to develop resistance to these metals and consequentially some other antimicrobials such as commonly used antibiotics. The possible percolation of these heavy metals from polluted soils into underground water and farmlands as well as the movement of autochthonous metal-resistant, pathogenic bacteria into such waters and their possible ingestion by unsuspecting animals and/or humans may result into development of diseases that may become difficult to treat as a result of resistance to these metals and native antibiotics used in the treatment of these diseases. Hence, the need for this research.

## Materials and Methods

### Sample collection

**Figure 1.**
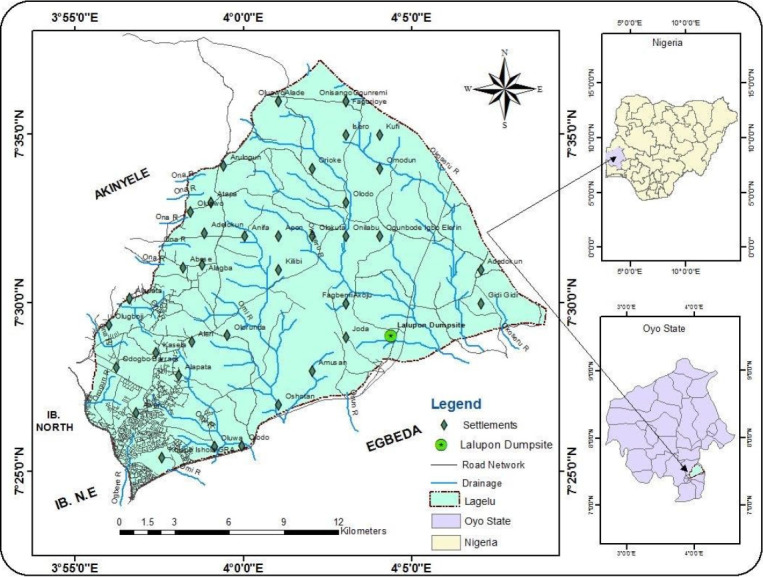
Map showing study area (Lalupon Battery Waste Dumpsite).

Top soil samples (10-15 cm) were collected from two different points A and B within a battery waste dumpsite (7° 29' 01" N, 4° 04' 23" E) at Lagelu local government area (Figure 1), while a third sample tagged as control was collected from natural soil without history of battery waste deposition away from the dumpsite using a soil auger into well-labelled, sterile resealable plastic bags in order to prevent contamination from external sources and transferred to the laboratory in icepacks for further analysis.

### Determination of heavy metal concentration of battery waste dumpsite soil

Concentrations of selected metals (Zinc, Cadmium, Lead and Nickel) were determined using the Atomic Absorption Spectrophotometry (Buck Scientific Model 210 VGP). Soil samples were freed of plants and organic matter by sieving through a 1.7 mm mesh, then dried at 90 °C for 24 hours in the oven. Soil samples (10 g) were digested with 5 mL of HCl, 5 mL of HNO_3_ and prepared for heavy metal analysis [20].

### Isolation of metal-tolerant bacteria

Following a ten-fold serial-dilutions of the samples, aliquots (1 mL) of the diluent were plated on nutrient agar incorporated with 50 µ g/mL of the test metal salts (NiSO_4_.H_2_O, CH_4_O_4_Pb.3H_2_O, CdCl_2_.2^1/2^H_2_O, ZnSO_4_.7H_2_O) using the pour plate technique. Prior to the incorporation of the metal salts, they were dissolved separately in distilled water and filtersterilised. Inoculated plates were incubated at 37°C for 24 hours and resultant colonies were purified via subsequent subculturing [21].

### Determination of minimum inhibitory concentration of the heavy metals against isolates

The minimum concentration of the heavy metals that inhibits the growth of the isolates was regarded as the minimum inhibitory concentration (MIC) and was determined using the agar diffusion technique as described earlier [21] by culturing each isolate on increasing concentrations (100-500 µ g/mL) of the different heavy metals. Isolate growing at one concentration was transferred to higher concentration and the MIC was noted when the isolate failed to grow [22].

### Identification of metal-resistant bacterial isolates

Isolates exhibiting multiple resistance (up to 400 µ g/mL) to the test metals were characterised morphologically and with appropriate biochemical tests [23] then, identified using the online Advanced Bacterial Identification System (ABIS) as described by Sorescu and Stoica [24].

### Antibiotics susceptibility profile of the isolates

The antibiotics susceptibility profile of each isolate was determined using the Kirby-Bauer disc diffusion technique [25] via swabbing standardised volumes of the isolates on freshly prepared Mueller-Hinton agar and the antibiotics discs of Ciprofloxacin (10 µ g), Amoxicillin (30 µ g), Imipenem (10 µ g), Trimethoprim/Sulfamethoxazole (25 µ g) and Tigecycline (15 µ g) were aseptically placed on the inoculated solid media. Petri-dishes were incubated for 24 hours at 37 °C, thereafter, the diameter of inhibition zones was measured and interpreted using standard guidelines [26].

### Data analysis

Means and standard deviations of replicate data during this study were determined and mean comparisons were conducted using Analysis of Variance (ANOVA) and independent samples t-tests where appropriate, with significance set at p< 0.05.

## Results

In this study, result shows the variation in heavy metal concentration of the soil sample. Lead has the highest concentration in soil sample in comparison with the other heavy metals, while Cadmium was present in trace amount and in the order Pb>Zn>Ni>Cd with concentrations of 2596.80 mg/kg, 1445.10 mg/kg, 50.05 mg/kg and 34.97 mg/kg respectively (Table 1).

**Figure 2.**
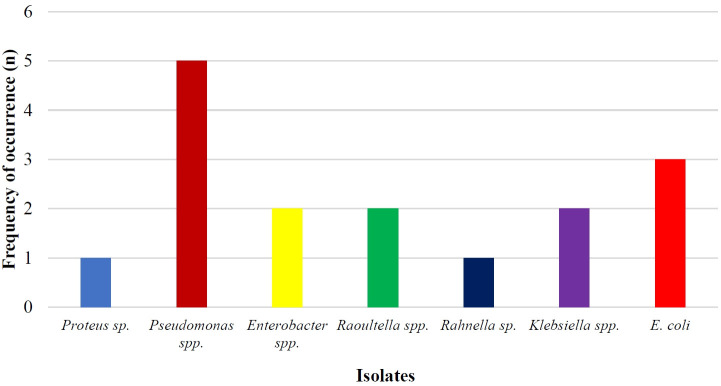
Distribution of metal-resistant bacteria isolated from metal-polluted soils.

As shown in Table 2, the total metal-resistant bacterial count in the soil samples ranged from 6.64 × 10^4^ CFU/g to 1.12 × 10^4^ CFU/g. It was observed that highest bacterial counts recorded for this study was 6.64 × 10^4^ CFU/g from Cadmium and the lowest bacterial counts was 1.12 × 10^4^ CFU/g from Nickel. In all, 16 isolates were recovered and identified as *Proteus* sp. (1), *Pseudomonas* spp. (5), *Enterobacter* spp. (2), *Klebsiella* spp. (2), *Escherichia* spp. (3), *Raoultella* spp. (2) and *Rahnella* sp. (1) (Figure 2). Of all the 16 isolates, 13 (81.25 %) showed multiple resistance to the heavy metals, that is, had an MIC of 400 µg/mL across the four test metals (Table 3).

The antibiotics susceptibility and resistance profile of the isolates are shown in Table 4. It was observed that 4 (30.8 %), 4 (30.8 %), 12 (92. 3 %), 2 (15.4 %) and 13 (100 %) were resistant to Trimethoprim/Sulfamethoxazole, Imipenem, Amoxicillin, Ciprofloxacin and Tigecycline respectively. In summary, 7 (43.75 %) of the isolates exhibited multidrug resistance (MDR) and the MDR profile is shown in Table 4.

## Discussion

Pollution of the environment in present times has been a significant environmental concern and is a consequence of human activities such as mining, modern agricultural techniques, industrialisation and urbanization. This study focused on the significance of heavy metal tolerance in selecting for antibiotics resistance in bacteria isolated from soil samples obtained from a battery waste site.

Soil samples obtained from the battery waste site revealed that the soil samples had a high degree of contamination by heavy metals. In this study, the order of abundance of the metals in the impacted site was Pb>Zn>Ni>Cd and was similar to previous reports [27]. It is noteworthy that the concentrations of these heavy metals in the soil samples exceeded the permissible limit for the selected heavy metals in soils as recommended by the World Health Organization [28]. Meanwhile, it was observed in this study that lead had the highest concentration in the soil samples. The presence of lead at the dumpsite which may be leached to the underlying soils of the dumpsite can be attributed to presence of lead-containing wastes being dumped in the study area as earlier established [27] in which high concentration of lead was reported in soil samples collected from an agricultural soil with history of battery waste deposition around Omilende area, Ibadan, Nigeria. More so, Alsbou and Al-Khanshman [29] reported high concentration of lead in soil collected on a street at Petra region, Jordan. This observation in this study may be due to the high concentration of lead as a major component in battery. The metals observed in this study are in such a high concentration that may have deleterious consequences such as lead poisoning, cancer, chest tightness and other respiratory illnesses.

Isolation of heavy metal-resistant bacteria is an indication of exhibition of resistance by these isolates. Sixteen metal-resistant bacteria were isolated from the soils and this may be hinged on their potential to adapt and tolerate high concentration of the heavy metals in the dumpsite. The isolation of heavy-metal resistant bacteria is in accordance with that of Yamina et al. [30] in which ten metal-resistant bacteria were isolated from a wastewater plant at Wadi El Harrach located in El Harrach city, Algeria. Onuoha et al. [31] reported high number of heavy metal-tolerant bacteria isolated from agricultural soil at Abakaliki, Ebonyi state, Nigeria while 137 heavy metal-tolerant bacteria were isolated from heavy metal polluted soil of different lands [32]. The presence of this high population of metal-tolerant bacteria in this study is evident of their tolerance to these heavy metals.

Isolates from this study identified as *Escherichia coli*, *Pseudomonas* spp., Bacillus spp., Enterobacter sp., *Raoultella* spp., *Rahnella* sp. and *Klebsiella* spp. have also been reported by several authors; Kolawole and Obueh [33] isolated *E. coli*, *Klebsiella* spp., and *Pseudomonas* spp. as heavy metal-tolerant bacteria from soil, water and plants in Utagba-Uno, Nigeria. Similarly, Akudo et al. [34] isolated *E. coli* and *Proteus* spp. as heavy metal-tolerant bacteria from heavy metal contaminated soils at Panteka stream. Koc et al. [35] also isolated *Raoultella* spp. as heavy metal-resistant bacteria from surface water while He et al. [36] reported that *Rahnella* spp. showed significant levels of tolerance to heavy metals, especially Pb, Zn, Ni and Cd from *Polygonum pubescens*. Sinegani and Younessi [32] reported that Pseudomonas, Bacillus, and Enterobacter isolated from heavy metal-contaminated soils showed high metal-tolerance this is in line with the observations from this study. The isolates are tolerant to heavy metals due to their ability to grow, proliferate and adapt to high level of metals in the sample sites.

Metal tolerance is important for microorganisms to survive metal-polluted environments. In the present study, 13 (81.25%) exhibited multiple tolerance to the heavy metals. Notably, all the isolates exhibited resistance to lead and this may be attributed to the high lead contamination in the study site and this may have arisen from the selective pressure on microorganisms as a result of continuous dumping of battery at the dumpsite, resulting in their ability to adapt to the high concentration of metal concentration. Similarly, high level of resistance to lead was reported by metal-tolerant bacteria isolated from soils contaminated with hydrocarbons and heavy metals [37]. More so, it was also observed that all the isolates did not grow at high concentration of nickel and this may be as a result of nickel toxicity in microorganisms [34].

An interesting finding from this study was that the metal-resistant bacteria were resistant to most antibiotics utilised. This observation corroborated the study of Hacioglu and Tosunoglu [38] in which high resistance of heavy metal-resistant bacteria isolated from an aquatic species at Kavak Delta to similar antibiotics was observed. Similarly, Omotayo et al. [39] reported that the heavy metal-resistant bacteria isolated from wastewater treatment plant exhibited resistance to two or more classes of antibiotics.

*Pseudomonas* sp., *Proteus* sp., Enterobacter sp. and Raoultella sp. isolated from this study exhibited co-selection to metals and antibiotics used in this study and this corroborates with the study of Yitayeh et al. [40] in which heavy metal-resistant *E. coli*, *Enterobacter* spp., *Proteus* sp., and *Klebsiella* spp. showed resistance to the classes of the antibiotics used in this study. Co-occurrence of heavy metals and antibiotics resistance in the bacterial isolates observed in this study have been reported by several authors; Heydari et al. [41] reported co-occurrence of metal and antibiotics resistance in bacteria isolated from agricultural soils in New Zealand. Similarly, Di-Cesare et al. [42] reported co-selection of bacteria isolated from freshwater at Lake Orta, Italy to heavy metals and antibiotics. Adekanmbi et al. [43] also reported co-existence of resistance to metals and antibiotics in *Vibrio* species isolated from fish ponds with and without antibiotics usage history. This observation is a major cause of concern for public health since illnesses caused by multidrug-resistant bacteria are difficult to treat and should they possess genes conferring such resistance, they may be transferred by different means to other bacteria present in their immediate environment, thereby conferring resistance to many classes of antibiotics as well.

## Conclusions

The bacteria isolated from the metal-polluted soils exhibited resistance to selected heavy metals they were tested against while also being resistant to different classes of antibiotics they were exposed to in this study. Therefore, the study has shown that persistent exposure of inherent bacteria isolated from soil in the battery waste dumpsite contributed to their development of resistance to the tested antibiotics. Should all these isolates seep into the underground water or come in contact with food and are ingested, they can lead to diseases or infections in human and will be difficult to treat as a result of their multidrug-resistant ability. This thereby increases disease burden and expenditure on seeking new drug alternatives for these antibiotic-resistant bacteria.

It is therefore recommended that adequate measures should be taken in disposal of battery wastes and dumpsites for such wastes should be erected in locations distant from human residence and contact to avoid contamination of food and drinking water by some autochthonous bacteria that may be pathogenic

## Figures and Tables

**Table 1.** Heavy metal concentration of contaminated soil samples collected from Lalupon

Metal	Sampling Point/ Metal concentration (mg/kg)			
	A	B	Control	WHO Permissible limit
Nickel	50.05 ± 0.07^a^	33.32 ± 0.57^b^	18.67 ± 0.09^c^	50
Cadmium	34.97 ± 21.26^b^	41.93 ± 0.10^a^	14.66 ± 0.29^c^	3
Zinc	1445.10 ± 0.05^b^	2160.3 ± 0.37^a^	0.35 ± 0.01^c^	300
Lead	2596.8 ± 4.48^a^	1060.7 ± 0.42^a^	11.20 ± 0.02^c^	100

Values are Means ± Standard Deviations of triplicate samplings. Means with different superscript alphabet across each row are significantly different from each other at p<0.05 using the Duncan Multiple Range Test of Analysis of Variance (ANOVA).

**Table 2.** Total metal-resistant bacteria count (TMRB) isolated from heavy metal-contaminated soil.

Sample	TMRB (×10^4^ CFU/mg)
Lead + Soil	3.65 ± 0.2^b^^c^
Zn + Soil	2.88 ± 0.12^c^
Ni + Soil	1.12 ± 0.12^c^
Cd + Soil	6.64 ± 0.28^a^

Values are Means ± Standard deviations of duplicate observations. Means with different superscript alphabet down the column are significantly different from each other at p≤0.05 using the independent samples t-test.

**Table 3.** Minimum Inhibitory Concentration (μg/mL) of selected heavy metals against the test isolates.

Isolate Code	Metals/ Concentration (μg /mL)			
	Zinc	Nickel	Lead	Cadmium
*Proteus* sp.	500	400	500	500
*Klebsiella* sp.	400	400	500	300
*Pseudomonas* sp.	400	400	500	400
*Enterobacter* sp.	400	400	500	300
*Klebsiella* sp.	500	300	500	400
*Pseudomonas* sp.	400	400	500	500
*Escherichia coli*	400	400	500	500
*Pseudomonas* sp.	500	400	500	500
*Pseudomonas* sp.	500	400	500	500
*Enterobacter* sp.	400	300	500	300
*Escherichia coli*	500	400	500	400
*Raoultella* sp.	400	400	500	300
*Rahnella* sp.	400	300	500	500
*Raoultella* sp.	500	400	500	500
*Escherichia coli*	400	400	500	200
*Pseudomonas* sp.	400	400	500	500

**Table 4.** Antibiotics resistance profile and Multidrug Resistance (MDR) index of isolates.

Isolate	Zone of Inhibition (mm)						
	SXT (25μg)	IPM (10μg)	AMC (30μg)	CIP (10μg)	TGC (15μg)	Resistance Profile	MDR index
*Proteus* sp.	12 (I)	14 (I)	6 (R)	20 (R)	0 (R)	AMC-CIP-TGC	0.6
*Pseudomonas* sp.	12 (I)	18 (I)	0 (R)	32 (S)	0 (R)	-	-
*Klebsiella* sp.	16 (S)	16 (I)	0 (R)	32 (S)	0 (R)	-	-
*Raoultella* sp.	12 (I)	20 (R)	0 (R)	28 (I)	0 (R)	IPM-AMC-TGC	0.6
*Escherichia coli*	20 (S)	10 (I)	0 (R)	30 (I)	2 (R)	-	-
*Pseudomonas* sp.	8 (R)	20 (R)	0 (R)	30 (I)	0 (R)	SXT-IPM-AMC-TGC	0.8
*Pseudomonas* sp.	6 (R)	0 (R)	0 (R)	30 (I)	0 (R)	SXT-IPM-AMC-TGC	0.8
*Rahnella* sp.	22 (S)	24 (S)	4 (R)	24 (I)	0 (R)	-	-
*Pseudomonas* sp.	12 (I)	18 (I)	2 (R)	30 (I)	0 (R)	-	-
*Pseudomonas* sp.	12 (I)	24 (S)	22 (S)	22 (I)	0 (R)	-	-
*Enterobacter* sp.	4 (R)	34 (S)	6 (R)	30 (I)	0 (R)	SXT-AMC-TGC	0.6
*Raoultella* sp.	0 (R)	14 (I)	4 (R)	20 (R)	0 (R)	SXT-AMC-CIP-TGC	0.8
*Escherichia coli*	24 (S)	20 (R)	1 (R)	30 (I)	20 (R)	IPM-AMC-TGC	0.6
% Resistance	30.8	30.8	92.3	15.4	100.0		

KEY: Ciprofloxacin – CIP, Imipenem – IPM, Trimethoprim/Sulfamethoxazole – SXT, Amoxicillin – AMC, Tigecycline – TGC, Sensitive (S), Intermediate (I), Resistance (R)
